# Structural plasticity of climbing fibers and the growth-associated protein GAP-43

**DOI:** 10.3389/fncir.2013.00025

**Published:** 2013-02-21

**Authors:** Giorgio Grasselli, Piergiorgio Strata

**Affiliations:** ^1^Department of Neurobiology, University of ChicagoChicago, IL, USA; ^2^National Institute of Neuroscience-Italy, University of TurinTurin, Italy

**Keywords:** climbing fiber, GAP-43, sprouting, atrophy, branching

## Abstract

Structural plasticity occurs physiologically or after brain damage to adapt or re-establish proper synaptic connections. This capacity depends on several intrinsic and extrinsic determinants that differ between neuron types. We reviewed the significant endogenous regenerative potential of the neurons of the inferior olive (IO) in the adult rodent brain and the structural remodeling of the terminal arbor of their axons, the climbing fiber (CF), under various experimental conditions, focusing on the growth-associated protein GAP-43. CFs undergo remarkable collateral sprouting in the presence of denervated Purkinje cells (PCs) that are available for new innervation. In addition, severed olivo-cerebellar axons regenerate across the white matter through a graft of embryonic Schwann cells. In contrast, CFs undergo a regressive modification when their target is deleted. *In vivo* knockdown of GAP-43 in olivary neurons, leads to the atrophy of their CFs and a reduction in the ability to sprout toward surrounding denervated PCs. These findings demonstrate that GAP-43 is essential for promoting denervation-induced sprouting and maintaining normal CF architecture.

## Introduction

Structural plasticity is limited in the central nervous system (CNS) of adult mammals, constituting a significant impediment to recovery from injuries such as those caused by trauma, stroke, and neurodegenerative and demyelinating diseases (Duffau, 2006; Wieloch and Nikolich, 2006; Landi and Rossini, 2010). Nevertheless, a relatively high degree of structural plasticity is retained by certain areas of brain, such as the cerebellum (Carulli et al., 2004; Cesa and Strata, 2009).

The cerebellar climbing fiber (CF), the terminal arbor of the olivo-cerebellar axons, has provided the first example, in the mammalian CNS, of individually observed fibers undergoing sprouting after brain injury (Rossi et al., 1991a,b). In 6-weeks-old Wistar rats, CFs normally encompass approximately 1000 μm of dendritic length and bear an average of 544 ± 23 varicosities that express the vesicular glutamate transporter VGLUT2 (Grasselli et al., 2011).

CFs constitute a suitable model that can be used to investigate axonal structural plasticity, based on their significant plastic potential and morphological hallmarks. In fact, they have a one-to-one relationship with their target Purkinje cell (PC). CFs undergo lesion-induced sprouting, activity-dependent remodeling, expansion of their area of innervation in response to an enlarged target territory, and regressive modifications after elimination of their target (Rossi and Strata, 1995; Strata and Rossi, 1998; Cesa and Strata, 2009).

## Structural plasticity of climbing fibers

Neurons differ widely in regard to their response to axonal injuries (Carulli et al., 2004; Dusart et al., 2005). For example, in the cerebellum, PCs respond to injury with little upregulation of plasticity-related genes in the cell body, no axonal regeneration after axotomy, and weak sprouting; most PCs survive, but they usually do not increase the expression of plasticity-related genes, except when the axotomy occurs near the cell body (Rossi et al., 1995; Bravin et al., 1997; Zagrebelsky et al., 1998; Wehrle et al., 2001; Morel et al., 2002; Gianola and Rossi, 2004). Further, axonal sprouting is limited and might be induced only following proper manipulation of intrinsic and environmental factors (Buffo et al., 1997, 2000; Zagrebelsky et al., 1998; Zhang et al., 2005, 2007).

In contrast, neurons in the inferior olive (IO) respond dramatically to axonal injury. The resection of olivo-cerebellar axons leads to the regression of the remaining stump and the death of many axotomized neurons in the IO during the first few weeks after injury (Buffo et al., 1998). Concurrently, olivary neurons upregulate several intrinsic factors, including nitric oxide synthase (NOS), c-Jun, JunD, the early growth response protein EGR1/Krox-24 (Rossi and Strata, 1995; Bravin et al., 1997; Buffo et al., 1998, 2003; Wehrle et al., 2001).

As a result of this upregulation and the high constitutive levels of growth-associated factors in olivary neurons, such as GAP-43, MARCKS, EGR-1/KROX-24, L1CAM, and PSA-NCAM in the olivary neurons (Kruger et al., 1993; Herdegen et al., 1995; McNamara and Lenox, 1997; Fernandez et al., 1999; Horinouchi et al., 2005), lesioned olivo-cerebellar axons can elongate and innervate their target PCs when an appropriate permissive environment is provided, such as neonatal Schwann cells that have been inserted at the site of axotomy (Bravin et al., 1997). Lesioned olivo-cerebellar fibers can also elongate into a transplant of embryonic cerebellum, where they innervate the grafted PCs, forming new CFs (Gardette et al., 1988; Rossi et al., 1995).

**Figure 1:**
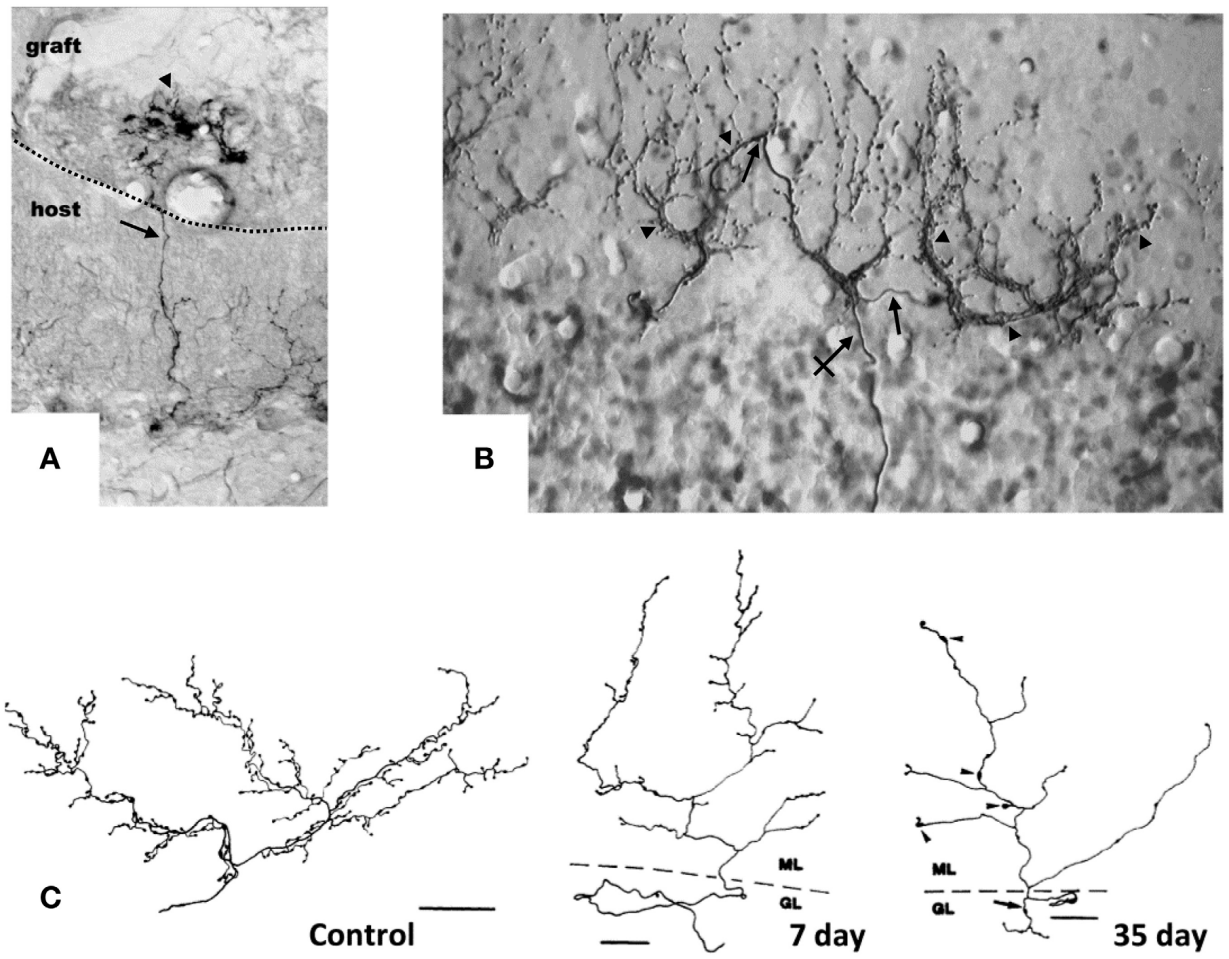
**Target-dependent structural plasticity of CFs. (A)** Sprouting of adult CFs 7 days following transplantation of embryonic cerebellum on to the surface of the adult cerebellar cortex. The sprouts (arrows) innervate PCs in the graft (arrowheads). Dotted line: host-graft border (Rossi et al., 1994, unpublished). **(B)** CF sprouting and innervation of adjacent PCs previously denervated by a partial lesion of the IO, shown 1 year after the lesion on a sagittal plane. From 1 olivo-cerebellar axon (crossed arrow), a CFs is formed with collateral branches (arrows) giving rise to new CF-like structures (arrowheads) innervating the adjacent PCs (Rossi et al., 1991b). **(C)** A control CF and atrophic modification induced in CFs 7 and 35 days after deletion of PCs. CFs were reconstructed using a camera lucida. GL, granular layer; ML, molecular layer. Scale bar: 30 μm, 25 μm, and 25 μm, respectively, for control, 7 and 35 days (Rossi et al., 1993). CFs were labeled by PHA-l axon tracing, PCs by anti-calbindin immunostaining.

The constitutive regenerative properties of olivo-cerebellar fibers render them responsive to axotomy and to the expansion or deletion of their target PC territory in the absence of direct cellular lesions. Grafting embryonic cerebellar tissue onto the surface of a non-lesioned host cerebellum leads to the formation of a minicerebellum whose PCs become innervated by collateral sprouting of intact host CFs that elongate across the pial barrier, likely under the influence of target diffusible factors. Consequently, they form new CF-like structures in the minicerebellum (Figure 1A) and on PCs that have migrated inside the host cerebellar parenchyma (Rossi et al., 1992) and establish functional synapses (Tempia et al., 1996).

Moreover, CFs can innervate and establish new functional synapses with additional nearby PCs, if the latter are deprived of their original CF innervation due to the neuronal degeneration of part of olivary neurons, selectively among pre-cerebellar nuclei, induced by intraperitoneal administration of the niacinamide analog 3-acetylpyridine (3-AP; Figure 1B) (Desclin and Escubi, 1974; Benedetti et al., 1983; Rossi et al., 1991a,b). Further, in neonatal rats (to 7–10 days after birth), olivo-cerebellar axons sprout and form long transcommissural branches to reinnervate the opposite hemicerebellum if it is denervated by transection of its peduncle (Sherrard et al., 1986). This form of transcommissural growth can be induced experimentally after development (30 days after birth) by infusion of exogenous BDNF (Dixon and Sherrard, 2006) or IGF-I (Sherrard and Bower, 2003) into the denervated hemicerebellum.

Conversely, if the target PC is deleted by neurotoxins, the CF arbor becomes atrophic, shrinking, and altering the shape of the varicosities (Figure 1C) (Rossi et al., 1993, 1995). Also, on blockade of electrical activity by tetrodotoxin or on inhibition of AMPA glutamate receptors with an infusing NBQX into the cerebellar parenchyma for 7 days, the varicosities of CFs decrease significantly in size, and fewer synaptic contacts are made with the spines of the proximal dendritic domain of PCs (Bravin et al., 1999; Cesa et al., 2007). These changes are attributed to findings that electrical activity mediates in the ongoing competition between the CF and parallel fibers (Cesa and Strata, 2009). Electrical activity of IO neurons also impedes the motility of the transverse branches of the CF that extend perpendicularly to the plane of the major structure of the fiber (Nishiyama et al., 2007).

## Extrinsic and intrinsic factors in CF plasticity: the function of GAP-43

The molecular determinants that induce, guide, and regulate CF elongation and innervation of PCs are only partially clarified. Like most mature CNS neurons, CFs can grow only in limited space that is devoid of extrinsic inhibitory influences, such as the cerebellar molecular layer, which lacks inhibitory myelin growth factors.

More is known about the intrinsic factors that confer highly plastic properties to CFs. The well-characterized plasticity of mature IO neurons is associated with high, constitutive expression of the growth-associated proteins GAP-43, EGR-1/KROX-24, MARCKS, L1CAM, and PSA-NCAM, and with the upregulation of c-Jun, JunD, Krox-24, and NOS in response to axonal lesions. However, the contribution of each factor is still not clear.

GAP-43 was one of the first of these proteins to be studied extensively and described for its abundance in axonal growth cones (Zwiers et al., 1976; Skene and Willard, 1981); thus it is used widely as a marker of axonal sprouting (Oestreicher et al., 1997). GAP-43 (also known as neuromodulin and B-50) mediates axonal growth, branching, and pathfinding during development. Mice that lack this protein have a low survival rate in the early postnatal period (Strittmatter et al., 1995; Maier et al., 1999). In humans, heterozygous chromosomal deletions comprising the locus for *Gap-43* gene (3q13.10–3q13.21) are linked to agenesis of the *corpus callosum* and severe mental retardation (Genuardi et al., 1994; Mackie Ogilvie et al., 1998).

GAP-43 plays a pivotal role not only during development but also in axonal remodeling in the adult brain. Its expression rises in several conditions that induce neuronal rewiring, such as the disruption of the neuronal networks due to pathological or traumatic lesions (Benowitz et al., 1990; Oestreicher et al., 1997; Buffo et al., 2003): it is upregulated in the motoneurons of dystrophin-deficient mice (*mdx* mice), a model of human muscular dystrophy, in which degeneration-regeneration events in muscle fibers are accompanied by remodeling of intramuscular terminal nerve fibers (Verzè et al., 1996), and after the induction of robust neuronal activity, for example due to seizure or electrical stimulation (McNamara and Routtenberg, 1995; Cantallops and Routtenberg, 1996; Miyake et al., 2002; Sharma et al., 2010).

Complex alterations in GAP-43 expression are frequently observed in human neuropathologies and their animal models, suggesting axonal damage or attempts of regenerative axonal sprouting. For instance GAP-43 expression declines in the frontal cortex and certain areas of the hippocampus in Alzheimer patients but is robust in association with senile-like plaques (Bogdanovic et al., 2000). Moreover, GAP-43 levels decrease in most lesions in the white matter of patients with multiple sclerosis and increase in some remyelinated white matter tracts (Teunissen et al., 2006).

**Figure 2:**
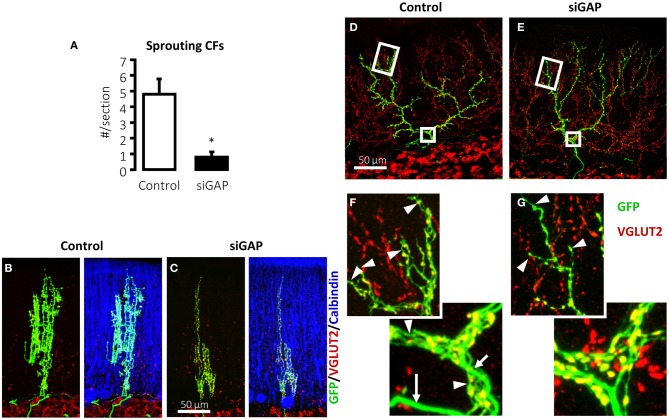
**Silencing of GAP-43 in CFs prevents its sprouting and induces atrophy. (A)** Sprouting in GFP-positive CFs, induced by a sub-total lesion of the IO, is dramatically reduced to nearly null levels in rats treated with GAP-43-silencing vectors (siGAP) 3 weeks before the lesion compared with controls as observed on coronal sections (*N* = 3 and 5 animals, respectively; ^*^p < 0.05; mean ± SEM). **(B,C)** The total extension of CFs that are still able to grow sprouts following GAP-43 silencing was also significantly reduced as assessed on coronal sections. **(D,E)** Representative confocal images of rat CFs in sagittal sections under normal conditions 3 weeks after treatment with control or silencing vectors. **(F,G)** Details of the most distal segment and first main branching point of CFs shown in **(D,E)**, showing a reduction in number and length of tendrils and consequent decrease in the density of varicosities. Arrows: thick axonal stalks; arrowheads: examples of tendrils [GFP, green; VGLUT2, red; calbindin, blue; modified from Grasselli et al. (2011)].

In several experimental conditions, GAP-43 overexpression *in vivo* increases axonal sprouting. In transgenic mice that overexpress GAP-43, motoneurons undergo axonal sprouting, even spontaneously in the absence of injuries, and increased sprouting after lesion. These mice experience prominent, spontaneous sprouting of mossy fibers in the dentate gyrus (Aigner et al., 1995). As discussed, when GAP-43 was overexpressed in PCs, their axons sprout profusely along their length and at their stump even at sites that are covered by myelin demonstrating that its overexpression is sufficient to induce sprouting in the absence of any injury and promote lesion-induced sprouting in PCs (Buffo et al., 1997; Gianola and Rossi, 2004). In a recent report, after silencing the expression of GAP-43 in IO neurons of juvenile wild-type rats using shRNA-expressing lentiviral vectors, their CFs were virtually unable to sprout in response to 3-AP-induced denervation of PCs (Figure 2A) (Grasselli et al., 2011). The few CFs that were, however, still able to sprout were significantly smaller than control fibers (Figures 2B,C). Because IO neurons are heterogeneous with regards to sprouting and gene expression after axotomy (Buffo et al., 2003), a more in-depth examination of the differences in CF morphology and their relationship to gene expression profiles of their neurons should provide greater insight into the function of the factors that regulate CF morphology.

GAP-43 is not only necessary for CF sprouting but plays also a crucial role for normal neuronal morphology in non-traumatic conditions. The mere silencing of GAP-43 destabilizes CF structure in the absence of any insult (Figures 2D–G) (Grasselli et al., 2011). Control CFs normally comprises a thick axonal stalk from which many thin collaterals emerge (namely tendrils), forming a net-like structure around the PC dendrite and bearing varicosities (Rossi et al., 1991b; Sugihara et al., 1999). Their structure has been examined quantitatively and a recent complete digital reconstruction shows that tendrils and distal branches are richer in varicosities (Brown et al., 2012).

On silencing GAP-43, CFs alter their structure, extending fewer tendrils along their proximal and distal portions (Figures 2D–G), quantified as a significant 17% reduction in the density of varicosities, as defined by their morphology and VGLUT2 expression. Further, the most distal portions of CFs, which have fewer tendrils and a thinner stalk, are affected by GAP-43 silencing, which shortens CF length by 33%. These data have been confirmed in 2–3-months-old mice (Grasselli et al., 2011).

Several lines of evidence support a model in which GAP-43 is needed for proper interaction of the axon with its target neuron and organization of the molecular machinery that supports axonal structures during axonal growth. In GAP-43 knockout mice, the axons of retinal ganglion cells fail to cross the optic chiasm properly (Strittmatter et al., 1995), instead assuming abnormal trajectories in the chiasm (Sretavan and Kruger, 1998). Moreover, these mice fail to form the anterior commissure, hippocampal commissure, and *corpus callosum* (Shen et al., 2002), consistently with the agenesis of the *corpus callosum* observed in patients who bear heterozygous chromosomal deletions comprising the *Gap-43* locus (Genuardi et al., 1994; Mackie Ogilvie et al., 1998).

In the hippocampus of transgenic mice that overexpress an inactive mutant form of GAP-43 that cannot be phosphorylated (with an amino acid substitution S42A), mossy fibers grow ectopically to their normal target layer, innervating the distal *stratum oriens* (Holahan et al., 2010). Notably, similar ectopic growth was observed in mice lacking the neuronal cell adhesion molecule NCAM (Cremer et al., 1997; Bukalo et al., 2004). L1CAM, another adhesion molecule that mediates commissural axon guidance (Kamiguchi et al., 1998; Demyanenko et al., 1999), regulates GAP-43 pathway, acting synergistically with it promoting axon growth and regeneration when overexpressed in PCs *in vivo* (Zhang et al., 2005).

L1CAM and NCAM are expressed at constitutively high levels in the IO (Horinouchi et al., 2005; Quartu et al., 2010), and GAP-43 responds to the NCAM pathway by being phosphorylated by protein kinase C (PKC), ultimately binding the actin filaments and other scaffolding proteins stabilizing their cytoskeletal complexes (Oestreicher et al., 1997; Riederer and Routtenberg, 1999; Mosevitsky, 2005; Denny, 2006; Chakravarthy et al., 2008; Ditlevsen et al., 2008). These findings suggests that, in CFs, GAP-43 synergizes with cell adhesion molecules to transduce target-dependent signals and stabilize the cytoskeleton.

In addition to maintaining of CF structure, GAP-43 might also govern the organization of the presynaptic terminal and, consequently, neurotransmitter release. When GAP-43 is silenced, CF varicosities undergo alteration in morphology, becoming rounder and larger compared with control varicosities (Grasselli et al., 2011), which are often irregularly shaped and smaller, mirroring the phenotype observed after blockade of AMPA receptor (Cesa et al., 2007). These changes might be related to GAP-43 calcium- and PKC-dependent control of the cytoskeleton.

Several studies have also established the involvement of GAP-43 in neurotransmitter release and synaptic plasticity (Dekker et al., 1989; Gianotti et al., 1992; Ramakers et al., 1995, 1999, 2000; Biewenga et al., 1996; Kantor and Gnegy, 1998; Routtenberg et al., 2000; Hulo et al., 2002; Denny, 2006; Powell, 2006; Holahan and Routtenberg, 2008; Holahan et al., 2010), reporting direct calcium-dependent interactions with components of the synaptic machinery, such as SNAP-25, syntaxin, and VAMP (Haruta et al., 1997), and with rabaptin-5, which regulate the recycling of synaptic vesicle (Neve et al., 1998).

Thus, increasing evidence suggest that GAP-43 has a double role in mature CFs in sustaining both injury-induced sprouting and the maintaining their structure under normal conditions, possibly by mediating cytoskeletal reorganization that is triggered by cell adhesion molecules and CFs interactions with their target. In addition GAP-43 appears to regulate the organization of CF presynaptic terminal and neurotransmitter release.

Emerging technologies, such as 2-photon microscopy and laser axotomy, will allow us to monitor cells during injury and repair in live mammalian brains and induce microscopic lesions, enabling us to determine the sequence of structural remodeling events that occur in single fibers after axotomy (Holtmaat and Svoboda, 2009; Allegra Mascaro et al., 2010).

### Conflict of interest statement

The authors declare that the research was conducted in the absence of any commercial or financial relationships that could be construed as a potential conflict of interest.
